# Strategic Analysis of Shiraz Medical Tourism Industry: A Mixed Method Study

**DOI:** 10.22086/gmj.v0i0.1021

**Published:** 2018-03-28

**Authors:** Mohammad Kazem Rahimi Zarchi, Alireza Jabbari, Nahid Hatam, Peivand Bastani, Tahereh Shafaghat, Omid Fazelzadeh

**Affiliations:** ^1^Health Care Services Management Department, School of Management and Medical Informatics, Shiraz University of Medical Sciences, Shiraz, Iran; ^2^Health Management and Economic Research Centre, Isfahan University of Medical Sciences, Isfahan, Iran; ^3^Department of Health Administration, School of Management and Information Sciences, Shiraz University of Medical Sciences, Shiraz, Iran; ^4^Health human resources. School of Management and medical Informatics, Shiraz University of Medical Sciences, Shiraz, Iran; ^5^Health Care Services Management Department, School of Management and Medical Informatics, Shiraz University of Medical Sciences, Shiraz, Iran; ^6^Health Tourism Office, Shiraz University of Medical Sciences, Shiraz, Iran

**Keywords:** Strategic Analysis, Medical Tourism, Health Tourism, SWOT

## Abstract

**Background:**

The connection between healthcare and tourism industries in many countries has created one of the largest service industries, i.e. "medical tourism industry" which brings significant benefits to the countries. The present study aimed to examine internal and external factors affecting Shiraz medical tourism industry along with the potential capabilities of the industry.

**Materials and Methods:**

This applied research is a mixed method study conducted in 2017 employing both qualitative and quantitative methods. The study population consists of all organizations involved in the medical tourism industry. Deductive qualitative content analysis was employed so as to determine the internal and external factors influencing Shiraz medical tourism industry. Furthermore, the SWOT technique was used to analyze the data obtained from individual interviews and meetings with expert panels.

**Result:**

Internal and external factors were classified into four main themes, namely strengths, weaknesses, opportunities and threats and ten sub-themes, of which five cases (FORMM) were related to internal factors (i.e. finance, production and products (operations), research and development, marketing and management) and five cases (STEPC) were associated with external factors: Socio-cultural, technological, economic, political and competitive. The matrix of the internal and external factors indicated an offensive zone for this industry.

**Conclusion:**

This industry can make use of the strengths and opportunities to confront threats and negative points through identifying internal and external factors and enjoy benefits such as job creation and revenue gains.

## Introduction

The association between healthcare and tourism industries in several countries has led to the creation of medical tourism industry which is one of the largest service industries [1-2]. Medical tourism is defined as individuals' travel to other countries to receive medical healthcare [3]. Factors highlighting the significance of medical tourism are the high cost of healthcare in industrialized countries, the increased ease of international trips, favorable exchange rate of currency in the global economy, rapid advances of medical technology and healthcare standards in most countries, internet access, inaccessibility of specific services in the country of origin, paucity of insurance coverage for certain services in the country of origin, waiting lists, confidentiality and privacy pertaining to services such as cosmetic and plastic surgeries, infertility treatment, trans-gender surgery, family visit, affordable international shipping costs, emergence of new companies and state supports [4-8]. Medical tourism services bring a significant benefit to the destination countries [2]; furthermore, and the trades associated with such services are recognizably profitable for the destination countries [2, 9]. In 2013, Bloomberg reported about 7 million worldwide trips taken for the purpose of medical tourism. Several studies have also demonstrated the increasing rates of medical tourism. Evidence points to the fact that Thailand, America, Malaysia, Singapore, India, Mexico, South Korea, Brazil, Taiwan, Turkey and Costa Rica have attracted the greatest number of medical tourists [10]. According to one study, it is also predicted that medical tourism market will reach $ 33 billion income by the end of 2019 [11].

Asia is one of the first policymakers in this area and is deemed as a competition in the market owing to its affordable and high quality healthcare services [11, 12]. In Asia, Thailand, Singapore and India are identified as the three major destinations for medical tourism and will account for more than 80 percent of Asian medical tourism market in the future [11].

Given its medical tourism advantages such as low cost, high quality health services, competent physicians and abundant natural attractions [6], Iran is planning to establish a facility to meet healthcare and medical needs. According to the vision plan, Iran intends to host 20 million foreign tourists by 2025, resulting in a $ 15 billion revenue gain [13], which policy can create myriad jobs and attract investment [6, 14]. Shiraz, a city located at the southern part of Iran, is being considered as a medical hub in the south and a potential destination for medical tourists due to its cultural and historical attractions, mild climate, and large number of public and private hospitals equipped with diagnostic and medical facilities and experienced physicians [15, 16]. However, evidence suggests that the city has failed to make use of all its potentials [16]. One of the reasons might be the lack of understanding concerning the internal and external factors affecting medical tourism in this city. The SWOT Technique (Strengths, Weaknesses, Opportunities and Threats) is an effective method employed to simultaneously analyze the strengths and weaknesses (internal factors) along with opportunities and threats (external factors)[17, 21]. This provides organizations with an opportunity to have a systematic and comprehensive understanding as to the factors influencing their environment [22].

Given the abovementioned points and with regard to the major benefits of medical tourism including revenue gain and job creation, the current study was carried out in order to identify the strengths, opportunities and the future of this industry in Shiraz, Iran.

## Materials and Methods

This is an applied study (done in 2017) using mixed methods of qualitative and quantitative analysis. The research setting is Shiraz (located south of Iran) and the population of the study consists of all stakeholders and organizations involved in medical tourism industry including the Governor-general of Fars Province, Shiraz University of Medical Sciences, Cultural Heritage, Handicrafts and Tourism Organization of Fars Province, Medical Council of the Islamic Republic of Iran (IRIMC), Ministry of Foreign Affairs office in Shiraz, Immigration and Passport Office, Shahid Dastgheyb International Airport, Chamber of Commerce, and Shiraz City Council. To specify the mission statement, vision and values for Shiraz medical tourism industry, this study made use of document analysis to identify the internal and external factors affecting the industry, and employed deductive qualitative content analysis to analyze these factors and SWOT technique through holding individual interviews and meetings with expert panels. The research was conducted in three main steps as follows:

### Step 1: Defining the Mission Statement, Vision and Values for Shiraz Medical Tourism Industry

So as to elucidate the mission, vision and values of the industry, the researcher studied the documents available in Iran and Shiraz obtained from the websites and archives of the Ministry of Health and Medical Education, Shiraz, Tehran University of Medical Sciences, Governor-general of Fars Province, Cultural Heritage, Handicrafts and Tourism Organization of Fars Province. To analyse the documents, Scott’s method, a four-stage approach, was employed. In this regard, the following points were examined after collecting documents concerning the Scott’s method: authenticity, credibility, representativeness and meaning of documents [23]. At this stage of the research, after analyzing the documents and specifying the draft mission statement, vision and values, they were submitted to 15 experts, stakeholders and managers along with a checklist containing several questions regarding a viable explanation of the statements [24]. They were asked to attend a panel meeting to evaluate the draft statement based on the aforementioned checklist. At the panel meeting, the mission, vision and values of Shiraz medical tourism industry were approved by all participants following a detailed discussion.

### Step 2: Identifying the Internal and External Factors Affecting the Medical Tourism Industry in Shiraz

To determine the internal factors (strengths and weaknesses) and external factors (opportunities and threats) affecting Shiraz medical tourism industry, the researcher conducted semi-structured in-depth interviews with 15 experts, stakeholders and managers including the following authorities: Deputy director of Tourism and Pilgrimage of Fars Province, chair of Shiraz University of Medical Sciences, head of the Health Tourism Office of Shiraz University of Medical Sciences, vice president of Tourism and Cultural Heritage Organization, head of Ministry of Foreign Affairs in Shiraz, president of the Medical Council in Shiraz, head of Immigration and Passport Office in Shiraz, director of Shahid Dastgheyb International Airport in Shiraz, representative of Shiraz Chamber of Commerce, two managers whose hospitals were successful in attracting medical tourists from the perspective of Shiraz University of Medical Sciences, two professors specializing in health services management with at least one article published on medical tourism and the directors of two private companies active in attracting medical tourists.

Based on the SWOT technique, four pivotal questions were posed. The interviews were carried out after finalizing the interview questions and making appointments with the interviewees. If necessary, guiding or exploratory questions (e.g. please explain more, and what does that mean) were adopted during the interviews in order to encourage the participants to keep talking and provide us with deeper access to information. Interviews were continued until data saturation was reached. During this step (Interview with experts), all interviews were transcribed and audio-recorded. Furthermore, the interviewees’ key points and phrases were pointed and discussed at the end of each question in order to ensure the accuracy of the researcher’s understanding. The interviews were coded and deductive qualitative content analysis was used to analyze them. In this regard, the analysis was first performed manually and then through NVIVO software version 10. It is worth noting that in order to ensure the accuracy of the collected data, the researcher made sure the four criteria were met during the interviews and analysis; these criteria were credibility (acceptance), dependability (consistency), conformability (corroboration) and transferability proposed by Lincoln and Cuba, and Strawbert and Carpenter [25, 26]. This part of the study was evaluated by two members of the research team who had no conflict of interest on the subject matter and had previous experience, knowledge and expertise in the field of qualitative studies.

### Step 3: Analysis of the Internal and External Factors Affecting Medical Tourism Industry in Shiraz using SWOT

Owing to the fact that we had to enter a maximum of 20 internal and 20 external factors in the SWOT matrix, so as to provide a more detailed analysis [24], the factors were ranked according to the comments put forth by five professors specializing in health services management and tourism management. To rank the factors, the researcher referred to the experts and asked them to complete a checklist with instructions so that the specified internal and external factors were scored from 1 to 4. After collecting the checklists, 20 internal and 20 external factors with the highest scores were selected and included in the final matrix to be evaluated. The matrix was also sent to the interviewees participating in the previous step (n=15). They were asked to score each factor from zero (unimportant) to 100 (very important) according to its significance rather than its current position such that the sum of the weight coefficients assigned to internal and external factors (separately) was equal to 100. The coefficient given to each factor reflected its relative significance in terms of its impact on the success of Shiraz tourism industry. The participants were further asked to assign a score ranging from one to four based on a factor’s current position in Shiraz tourism industry and the effectiveness of the strategies adopted by this industry in response to the opportunities, threats, weaknesses and strengths. Finally, the weighted score of the industry (multiplying the relative importance by the score of each factor) was 1 (minimum) and 4 (maximum). The mean score was 2.5 and values higher than 2.5 represented the use of strengths and opportunities, while those below 2.5 indicated the industry’s failure to use strengths and opportunities to restrict weaknesses and threats. SWOT matrix was drawn once all internal and external factors were obtained and scored.

The type of strategy to be adopted by Shiraz Medical Tourism Industry in the future was specified using scores achieved from the analysis of the current situation.

Further considered in the present paper were certain ethical issues, such as permission and recommendation letter from Research and Technology Department of Shiraz University of Medical Sciences, oral and written informed consent during the study and interview recording , respecting the privacy of individuals, confidentiality of information, and granting the participants a right to withdraw at any stage from the study

## Result

Tables-1, 2 and 3 show the mission statement, vision and values pertaining to Shiraz Medical Tourism Industry. In general, this industry has to use its facilities and potentials to attract 50,000 tourists by the end of 2019.

**Table-1:** Vision Statement of Shiraz Medical Tourism Industry

With God’s help, Shiraz Medical Tourism Industry intends to use its own potentials to attract 100,000 medical tourists by 2019 through the development of medical and tourism services based on the needs of the tourists.

**Table-2:** Mission Statement of Shiraz Medical Tourism Industry

Shiraz Medical Tourism Industry is operating consistent with the objectives of Shiraz University of Medical Sciences and Cultural Heritage, Handicrafts and Tourism Organization of Fars Province. In addition to providing high quality, various and distinctive tourism and medical services including ophthalmology, hair transplantation, and cosmetic surgery, this industry is operating with experienced and famous physicians, updated medical equipment, travel agencies, historical, natural and religious sites accompanied with respect for patient and citizens’ rights, especially for medical tourists in the Persian Gulf and Central Asia. The major objectives of this industry are economic growth along with strengthening the spirit of solidarity, considering the human values ​​and Islamic-Iranian culture. In this regard, the presence and participation of all employees and stakeholders is of utmost importance.

**Table-3:** Values of Shiraz Medical Tourism Industry

Emphasis on justice	Aligning the objectives of this industry with the Islamic Republic of Iran’s 20-year vision
Commitment to the rules and regulations of the Islamic Republic of Iran	Respect for medical tourists
Using appropriate updated equipment	Emphasis on Islamic and Iranian culture
adhering to moral values	Use of collective wisdom and participation of managers and policy makers
Emphasis on the updated knowledge of Medicine and Tourism Day	Responsibility and accountability
Addressing the tourists’ complaints as soon as possible and compensating for the losses incurred for medical tourists	Services tailored to the needs of tourists and not providing tourists with a surplus of or poor standard services
Ensuring the privacy of medical tourists’ information	Providing the tourists with transparent billing and documentation
Adhering to the announced tariffs	Attention to the cultural and religious beliefs of the tourists at the time of service provision

Interviews were performed with experts, stakeholders and managers, resulting 20 internal and 20 external factors in the form of four general themes and ten sub-themes (Tables-4 and 5). As shown in Tables-4 and 5, the internal factors (strengths and weaknesses) include 5 subthemes (FORMM) (i.e. finance, production and products (operations), research and development, marketing and management), and external factors (opportunities and threats) contain five subthemes (STEPC), namely “socio-cultural, ecological and environmental”, “technological”, “economic”, “political/governmental and legal”, and “competitive”, each separately discussed below.

**Table-4:** Internal Factors Affecting Shiraz Medical Tourism Industry

**Weighted score**	**Score**	**Index**	**Main Internal Factors**	**Subthemes**	**Themes**
17.36	3.33	5.21	Low prices of tourism services and medical services in Shiraz compared to the Persian Gulf countries	Finance	*Strengths*
17.12	3.33	5.14	Providing more diverse and distinctive healthcare services compared to the Persian Gulf countries with short-term waiting list	Operations	
23.07	3.83	6.02	Presence of experienced, skilled and well-known medical staff including physicians in Shiraz		
15.24	2.66	5.71	Establishment of new hospitals in Shiraz		
17.17	3.33	5.33	High quality of tourism and medical services in Shiraz compared to the Persian Gulf countries		
17.17	3.17	5.42	Large number of well-known hospitals and updated medical equipment in Shiraz		
16.08	3.33	4.82	Internal accreditation hospitals according to IPD standards released by the Ministry of Health and Medical Education to attract medical tourists	Research
14.64	3.17	4.62	Presence of dealers in attracting medical tourists in terms of marketing	Marketing
22.70	3.83	5.92	Accepted background of Shiraz and Shiraz University of Medical Sciences over the past years	
16.10	3.33	4.83	Presence of convenient transportation, hotels, residential areas and many restaurants in the city	Management
21.34	3.83	5.57	Immediate Visa issuance at Shiraz Shahid Dastgheyb International Airport	
18.62	3.50	5.32	General Governor of pilgrimage and tourism in Fars Province and health tourism committee addressed by him	
8.6	1.67	5.16	Non-transparency of prices related to medical services and lack of information packages on tariffs	Finance
5.50	1.33	4.13	Absence of the international accreditation of hospitals	Research	*Weaknesses*
8.17	1.67	4.90	Lack of management, especially in ​​marketing and analysis of rivals	Marketing	
6.77	1.33	5.08	Paucity of appropriate advertising, notifications, updated hospital websites and online admission		
7.79	1.67	4.67	Lack of trained personnel to communicate and deal with medical tourists	Management	*Weaknesses*
5.75	1.50	3.83	Lack of coordination, common policies and specified strategies among the multiplicity of decision-making centers		
4.84	1.50	3.23	Lack of trust in the private sector to enter the industry		
6.72	1.50	4.48	Lack of statistics related to medical tourists		
272		100	Sum		
2.72			Total score		

**Table 5:** External Factors Affecting Shiraz Medical Tourism Industry

**Weighted score**	**Score**	**Index**	**Main Internal Factors**	**Subthemes**	**Themes**
22.64	3.83	5.91	Adjacency to the Persian Gulf states, with which we share a common religion and a sense of cultural affinity and the more solid ties among people form Shiraz living in these countries	Socio-cultural	*Opportunities*
17.94	3.17	5.67	Geographical location, mild climate and historical, religious and natural sites in Shiraz		
13.13	2.67	5	Foreign students in Shiraz and their familiarity with Shiraz healthcare services	Technology	
16.61	3.17	5.24	Relative improvement of the diplomatic relations of Iran, stable inflation in the country over the recent years, and the facilitation of the arrival of tourists to Shiraz	Political	
26.46	4	6.61	Iran’s national security, hence Shiraz’s		
11.86	2.50	4.74	The lack of legislation prohibiting the advertisement of medical services in foreign countries		
14.80	2.83	5.22	The poor quality of service delivery in other countries and aging in countries with weak health systems	Competitive	
15.73	3	5.24	Lack of security in rival countries regarding healthcare services		
19.27	3.33	5.78	Shiraz fame in the international communities		
17.22	2.83	6.08	Expensive medical and tourism services in competitor countries		
8.85	2.67	3.32	Immigration of experienced medical professionals to other countries	Socio-cultural	
11.22	2.33	4.81	Lack of trust in Iranian planes	Technology	
11.05	2.50	4.42	Expensive medical services following the implementation of the Relative Tariffs Services	Economic	
15.40	3	5.13	Weaknesses in Iran’s international relations with certain countries, political instability at the Ministry of Foreign Affairs in different governments, uncertainty as to entering the medical tourism industry, economic and banking sanctions against Iran	Political	*Threats*
11.75	2.17	5.42	Legal, welfare, and religious restrictions and regional unrest for tourists, especially medical ones		
12.73	2.67	4.77	Negative publicity in other countries as to Iran’s security	Political	*Threats*
9.89	2.33	4.24	The existence and activities of non-specialists (intermediaries/dealers)		
10	2.50	4	Seasonality of tourism in Shiraz		
11.87	2.17	5.48	European and Asian countries advertising their medical services for the Persian Gulf countries, creating a strong marketing competition	Competitive	
6.22	1.67	3.73	Progress of the Persian Gulf countries in the field of health		
282		100	Sum		
2.82			Total score		

### Strengths

On the basis of the interviews, the most important factors classified as strengths are “distinct and diverse services “, “reputed and experienced medical staff “, “quality of services”, “low-price services” and “Shiraz accepted background in the field of medical sciences”. Concerning “distinct and diverse services”, one of the interviewees noted: “Some surgical procedures in Shiraz are exclusive. In other words, there is certain exclusive equipment which makes the Arabs come to Iran and Shiraz”.

Regarding “reputed and experienced medical staff”, interviewees 1, 2 and 11, respectively stated: “One of the great potentials of Shiraz is its known and experienced physicians, which attracts medical tourists”, “ The most significant strength of medical tourism in Shiraz is the presence of some well-known, expert, and unique specialists across the Middle East”, and “ The first strength of Shiraz Medical Tourism is its being a brand, i.e. having a long time reputation of hosting skillful and well-known physicians , which makes the patients, especially those from Arabic countries, come here.”

In terms of “quality of service” and “low-price services”, interviewees 1 and 2 claimed: “Our services are less expensive comparisons to the Persian Gulf countries” and “Over the recent decades, there has been much emphasis, though still not quite scientific, on quality improvement.

We received a model template updated annually, rendering the quality of our services better.” Regarding “accepted background of Shiraz in the field of medical sciences”, interviewee 5 noted: “Apart from medical tourism, Shiraz has always been attracting tourists. This makes the tourists who travel to Shiraz spread its fame more.”

### Weaknesses

Based on the interviews, the weaknesses are “non-transparency of the prices pertaining to medical services and the paucity of information package on tariffs”, “lack of appropriate advertising, notifications and updated hospital websites and online admission”,” the void of coordination, common policies and specified strategies among the many decision-making centers “, and “lack of trained personnel to communicate and deal with medical tourists”. As far as the first three weaknesses are concerned, certain interviewees asserted: “Perhaps, the most recognizable disadvantage is that there exists no modern marketing technique for medical tourism in Shiraz, meaning that we have no active and effective medical tourism companies to do marketing for our medical tourism in other countries” or “We have no already prepared medical tourism packages. In fact, there are no detailed packages associated with Shiraz medical facilities. The packages would have contained websites, DVDs and manuals in the source languages. We are not good at marketing and we have no up-to-date and appropriate websites. A preponderance of hospital websites are either inactive or merely a simple demo” or “Foreign tourists are oblivious to the costs in these hospitals”. As concerns “the void of coordination, common policies and specified strategies among the many decision-making centers”, interviewees 5, 6 and 13 respectively asserted: “The most significant weakness is the chaos in medical tourism. No effectual management is applied in this field and it is unclear who is in charge “,” I think the most important factor is the lack of a trustee. It is not clear who the owner of medical tourism is” and “The main obstacle is the multiplicity of decision-making centers and the lack a coordinated and aligned plan for these centers. In other words, each facility wants to be in charge.” In terms of “the lack of trained personnel to communicate and deal with medical tourists”, the interviewees mentioned: “ Other weaknesses can be the lack of appropriate behaviour towards Iranian and foreign patients on the part of the personnel. Yet another weakness in this field is the personnel’s unfamiliarity with English and Arabic languages and other cultures. “

### Opportunities

“Adjacency to the Persian Gulf states, with which we share a common religion and a sense of cultural affinity and the more solid ties among people form Shiraz living in these countries”, “geographical location, mild climate and historical, religious and natural sites in Shiraz” and “lack of security in rival countries in as far as healthcare services are concerned” are among the opportunities extracted from the interviews. Interviewee 12 said: “One of the opportunities we enjoy is the historical and inseparable connection with Arab countries, which if better utilized, more favorable results would be obtained. Many residents of Arabic countries are Iranians particularly from Shiraz who have kept their connection with Shiraz” or “Another opportunity is the religious commonalities existing among nations of these regions. For instance, it is important for many Persian Gulf citizens to receive healthcare services in a country where the doctors are Muslim, especially Shiite”. Interviewee 3 stated: “I think the most important opportunity is the lack of security in our rival countries and those in the region, which would result in the attraction of medical tourists”. Interviewee 14 mentioned: “One of our opportunities is the current peaceful situation in the region. All our competitors, at least in the Middle East, are currently involved in war and civil disturbances and terrorism, unfortunately “.

### Threats

Based on the results of the interviews, the factors classified as threats to the medical tourism industry in Shiraz are “existence and activities of non-specialists (intermediaries) attracting medical tourists”, “seasonality of tourism in Shiraz”, and “expensive medical services following the implementation of relative tariffs on Services”. Interviewee 3 said:” One of the threats is dealers who have grown because of the legal gaps and lack of a unified policy and coordination among organizations. Nothing serious has been done in this regard.” Interviewee 7 stated: “One of the threats in this area is the arrival of more tourists from spring to the end of summer. We could almost certainly say that we have no medical tourism to manage from October to March “. Interviewee 12 mentioned: “One of the threats we discuss is the competitiveness of the service costs. In terms of costs, Shiraz is not competitive as regards many services. One of the major factors influencing medical tourism is the cost. He who does not care about prices travels to Europe and America, while for those who travel to Shiraz, price matters. When the cost difference, in comparison with western countries, is not significant, we would certainly lose our position in the long run”. Of the 20 factors identified in the evaluation matrix of the internal factors, 12 were specified to be the strengths and 8 were deemed as weaknesses in Shiraz medical tourism industry. The total score of internal factors affecting Shiraz medical tourism in the evaluation matrix was estimated to be 2.72, meaning the strengths of internal factors impacting medical tourism are more than its weaknesses (Table-4). Additionally, of the 20 factors identified in the evaluation matrix of the external factors, 10 were defined as opportunities and 10 as threats. The total score of external factors was estimated to be 2.82 which means that this city could have partly benefited from factors creating opportunities or position and avoided those causing threats (Table-5).

**Figure-1:**
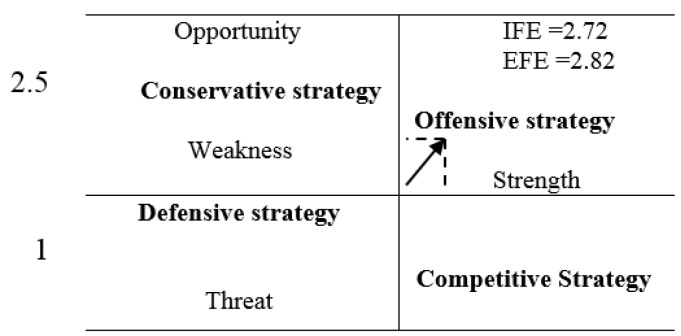
Shiraz Medical tourism strategies

As shown in Figure-1, and on the basis of the scores obtained in the matrix of internal and external factors, Shiraz medical tourism industry is in an offensive zone in terms of future strategies.

## Discussion

The results of this study showed that the most important factors affecting Shiraz medical tourism industry are low prices and high quality services, highly qualified medical staff, diverse and distinct services and immediate visa issuance. The findings are consistent with the results obtained by Delgoshaei (2012), Asadi (2011), Jabbari (2013) and Rerkrujipimol (2011). In their research, they indicated the importance of low prices, high quality, ease of visa issuance and well-known and experienced medical staff in attracting medical tourists [27-30]. Our results are also in a similar vein with Avinash’s (2008) study where the quality of medical care prior to, during and following treatment was demonstrated as a current challenge in medical tourism of developing countries [31]. In this regard, it can be claimed that the main factor attracting medical tourists is quality health care by physicians known at international levels and at lower prices so that the tourists not only receive effective treatment but also enjoy a decrease in their costs. The results of this study also showed that the medical and tourism opportunities, facilities and equipment are one of the key factors affecting the development of medical tourism. Similarly, Jabbari (2013) necessitated the development of facilities and infrastructures for medical centers and hospitals and the promotion of medical technology and equipment for the progress of the medical tourism industry [16]. On the other hand, Delgoshaei (2012), Salimpour (2007), Jabbari (2009) and concluded that intersectional coordination is of paramount importance in the medical tourism industry with respect to its multidimensionality, involving all parties such as the health sector, tourism sector, private sector, Ministry of Foreign Affairs, Passport Office, and insurance companies, all of which are to act coordinately [27-29]. In this study, lack of coordination and a common policy and strategy among the multiplicity of decision-making centers, is deemed as a weakness in the medical tourism industry in Shiraz; coordination among stakeholders is one of the most important challenges of the industry.

In addition, the current study revealed that promotion is yet another significant factor poorly dealt with in Shiraz. Promotion mechanisms such as preparing brochures, pamphlets, clips, etc. Must be adopted to introduce the potentials of the industry to other target countries. Ayoubian (2013) noted that the media plays a vital role [32]; advertising the capabilities of the country in the field of health care services through cross-border media could be effective in attracting medical tourists [32]. He recommended advertising the capabilities and treatment and welfare facilities of medical centers in the international media, providing information on the embassies, presenting brochures and pamphlets to foreign tourists, participating in international exhibitions, creating information portables, providing photos of the treatment centers, and hanging posters at the entrance and exit of international airports [32]. In his study, Afshani (2010) suggested that one of the major weaknesses of Iran medical tourism industry is the lack of convenient and professional information provision, particularly the gaps in the e-services of hospitals, leading to obliviousness of the actual potentials of international health services and their use [33]. Johnston (2016) also highlighted the role of promotion as the most important factor in attracting medical tourists and development in medical tourism [34]. Jabbari (2013) listed the elements influencing the medical tourism industry as follows: Reputable and well-known physicians, provision of immediate services, excellent facilities, high-tech medical equipment, and international accreditation [16]. Another study examined the current status of medical tourism in Iran and came to the conclusion that Iran is facing challenges in terms of effective support from the government, inter-organizational cooperation and coordination at macro and operational levels, health services providers with an international reputation, and an integrated promotion and marketing; if Iran intends to have an appropriate share of medical tourism market, it has to take certain measures with regards to these challenges [35].

In the current study, these were also mentioned as the main challenges in the way of Shiraz medical tourism industry. The results of this research are also in line with the study conducted by Ghanbari (2014) on determining an appropriate strategy for the medical tourism industry (in Ahvaz) where based on the analysis of internal and external factors and scores obtained from experts, the proposed strategy was in an offensive zone [36]. In the present research, on the other hand, the strengths and opportunities mentioned for the development of Shiraz medical tourism industry were higher than weaknesses and threats.

Accordingly, the formulation of development strategies for Shiraz medical tourism industry must be a priority. In spite of the immense potentials of Shiraz in attracting medical tourists, no serious measures has been taken in this regard, one of the most important reasons of which is the lack of coordination among the organizations involved in this industry. Identifying the strengths, weaknesses, opportunities and threats in the industry can play a critical role in coordinating the organizations involved. With regards to the fact that this industry is located at the offensive zone, it should benefit from its capabilities more effectively and promote the attraction of medical tourists, which ultimately leads to job creation and revenue gains.

## Conclusion

Given the immense potentials of Shiraz in attracting medical tourists, unfortunately, no serious measure has been taken in this regard yet. One of its most important reasons is the lack of coordination among the organizations involved in this industry. Identifying the strengths, weaknesses, opportunities and threats of this industry can play a critical role in coordinating the involved organizations. With regard to the fact that this industry is located at the offensive zone, it should take benefits of its capabilities more effectively to promote attracting medical tourists and consequently job creation and revenue gains.

## Conflict of Interest

The authors have no conflicts of interest
